# Sweat it out? The effects of physical exercise on cognition and behavior in children and adults with ADHD: a systematic literature review

**DOI:** 10.1007/s00702-016-1593-7

**Published:** 2016-07-11

**Authors:** Anne E. Den Heijer, Yvonne Groen, Lara Tucha, Anselm B. M. Fuermaier, Janneke Koerts, Klaus W. Lange, Johannes Thome, Oliver Tucha

**Affiliations:** 1 Department of Clinical and Developmental Neuropsychology, Faculty of Behavioural and Social Sciences, University of Groningen, Grote Kruisstraat 2/1, 9712 TS Groningen, The Netherlands; 2 Department of Experimental Psychology, University of Regensburg, Universitätssraße 31, 93053 Regensburg, Germany; 3 Department of Psychiatry and Psychotherapy, University of Rostock, Gehlsheimer Straße 20, 18147 Rostock, Germany

**Keywords:** Physical, Exercise, Children, Adults, ADHD, Cognition, Behavior

## Abstract

As attention–deficit/hyperactivity disorder (ADHD) is one of the most frequently diagnosed developmental disorders in childhood, effective yet safe treatment options are highly important. Recent research introduced physical exercise as a potential treatment option, particularly for children with ADHD. The aim of this review was to systematically analyze potential acute and chronic effects of cardio and non-cardio exercise on a broad range of functions in children with ADHD and to explore this in adults as well. Literature on physical exercise in patients with ADHD was systematically reviewed based on categorizations for exercise type (cardio versus non-cardio), effect type (acute versus chronic), and outcome measure (cognitive, behavioral/socio-emotional, and physical/(neuro)physiological). Furthermore, the methodological quality of the reviewed papers was addressed. Cardio exercise seems acutely beneficial regarding various executive functions (e.g., impulsivity), response time and several physical measures. Beneficial chronic effects of cardio exercise were found on various functions as well, including executive functions, attention and behavior. The acute and chronic effects of non-cardio exercise remain more questionable but seem predominantly positive too. Research provides evidence that physical exercise represents a promising alternative or additional treatment option for patients with ADHD. Acute and chronic beneficial effects of especially cardio exercise were reported with regard to several cognitive, behavioral, and socio-emotional functions. Although physical exercise may therefore represent an effective treatment option that could be combined with other treatment approaches of ADHD, more well-controlled studies on this topic, in both children and adults, are needed.

## Introduction

Attention–deficit/hyperactivity disorder (ADHD) is one of the most common neurodevelopmental disorders diagnosed in childhood, with symptoms continuing into adolescence and adulthood [American Psychiatric Association (APA) 2013]. The vast consequences of ADHD on individuals’ lives stress the importance of proper and scientifically based treatment options. In the past decades, the prescription of stimulant medication has substantially increased and has become a matter of social debate (Cortese et al. 2013). In this literature review, we systematically reviewed the potential benefits of an alternative or additional treatment option for ADHD, namely, physical exercise.

### Symptoms and cognition of patients with ADHD

The Diagnostic and Statistical Manual of Mental Disorders (DSM-5) describes inattention, impulsivity, impaired inhibition, and hyperactivity as the most common ADHD symptoms [American Psychiatric Association (APA) 2013], giving rise to problems in everyday life, social, academic, and occupational domains (Gapin et al. 2011; Hoza et al. 2005; Loe and Feldman 2007; Uekerman et al. 2010). Furthermore, more specific deficits in higher cognitive functions (executive functions) have often been demonstrated in ADHD (Barkley 2004). These functions facilitate self-monitored and self-regulated goal-directed behavior (Gioia et al. 2001). For example, deficits have frequently been reported in working memory, response inhibition, cognitive flexibility, risk taking, and planning (Barkley, 1997; Groen et al. 2013; Pliszka et al. 2006; Semrud-Clikeman et al. 2008; Toplak et al. 2009). Impairments of cognitive flexibility, in particular, may play an important role in behavior regulation and may underlie the often seen perseverative, non-adaptive, and dysregulated behavior in children with ADHD (Lezak 2004).

### Advantages and disadvantages of pharmacological treatment of ADHD

For several decades, stimulant medication (mainly methylphenidate, MPH) has constituted the most frequently applied treatment of ADHD (Huang and Tsai 2011; Pliszka et al. 2000; Tucha et al. 2006). On a neurobiological level, MPH increases dopamine and norepinephrine levels in the prefrontal cortex (PFC) (Oades et al. 2005; Pliszka 2005) and generally enhances central nervous system (CNS) activity (Semrud-Clikeman et al. 2008). On a behavioral level, an increase in alertness and a decrease in antisocial behaviors (e.g., aggression) and impulsivity have been reported following stimulant drug treatment (Rhodes et al. 2006; Semrud-Clikeman et al. 2008; Wilson et al. 2006). Stimulants have also been shown to positively affect various aspects of cognition, including domains of attention (e.g., mental alertness), executive functioning, memory, and visuospatial functioning as well as cognition related to self-regulation (Huang and Tsai 2011; Rhodes et al. 2006; Semrud-Clikeman et al. 2008; Tucha et al. 2006; Wilson et al. 2006). Furthermore, MPH was found to improve quality of life and academic achievement (Huang and Tsai 2011).

Despite the existence of well-elaborated evidence-based guidelines (NICE Guidelines on ADHD 2008) and the fact that efficacy and safety of stimulants have been proved in a considerable number of studies and meta-analyses (e.g., Maia et al. 2014; Rubia et al. 2014; Thapar and Cooper 2016), uncertainties still exist concerning stimulant drug treatment for ADHD. These include sub-optimal treatment in clinical practice (Hodgkins et al. 2013), uncertainty about the physical safety and potential long-term and side effects of stimulant medication (Biederman et al. 1991; Buitelaar and Medori 2010; Dela Pena and Cheong 2013; Owens et al. 2000; Sonuga-Barke et al. 2009; Stein et al. 2003; Taylor et al. 2004; Van der Heijden et al. 2006; Wilens et al. 2008) and a relatively high non-response rate of 10–25 % (Banaschewski et al. 2006; Taylor et al. 2004). Besides pharmacological approaches to ADHD treatment, there are non-pharmacological treatment options, such as cognitive trainings, neurofeedback, and behavioral therapy, diets, and combined interventions. Studies on these alternative treatments are relatively scarce, but do show improvements in ADHD symptoms with small to medium effect sizes (see for a meta-analysis: Sonuga-Barke et al. 2013). However, many of these studies are flawed with methodological weaknesses, and the beneficial effects often disappear when controlling for blinded assessment.

### General effects of physical exercise in relation to ADHD

Recently, physical exercise has been proposed as an alternative or additional treatment for ADHD. Particularly for children, physical exercise has been raised as a safe and effective ADHD symptom management method, having beneficial effects on both cognition and behavior (Gapin et al. 2011). Physical exercise can be subdivided into several categories. First, *cardio exercise* raises the heart rate, stimulates perspiration, and makes one out of breath. This category comprises activities such as running, cycling, dancing and treadmill exercise. Cardio exercise has been associated with several ameliorations of physical and cognitive functions (e.g., executive functions) in healthy children (Best 2010; Hill et al. 2011). The second category is *non*-*cardio exercise* which is tranquil, e.g., yoga and tai chi, and has also been linked to both physical and cognitive gains in healthy children (Field 2012). Another categorization of exercise is based on the duration of effects, which can be classified as either *acute* (i.e., effect measurable shortly after the physical activity) or *chronic* (i.e., effects extending after a considerable period of rest).

Well-known general positive effects of exercise include the enhancement of overall physical fitness, growth and density of bone minerals, and the reduction of obesity and inflammation (Field 2012). In addition, positive effects of exercise on psychological and cognitive functions have been described (Hill et al. 2011; Hillman et al. 2008). Sibley and Etnier (2003) observed acute as well as chronic effects of various cardio and non-cardio exercises on perceptual skills, intelligence, academic achievement, developmental level and performance on verbal and mathematic tests in children and adolescents (4–18 years). Furthermore, improvements of executive functions of children have been demonstrated following cardio exercise (Best 2010). These benefits in executive functions were strongest in exercise types that demand cognitive engagement, entailing cooperation awareness, anticipation, task-demands and team-sport strategic thinking, compared with exercise types that do not require such cognitive involvement (Best 2010).

It is assumed that the mechanisms by which physical exercise provokes beneficial psychological and/or cognitive effects are an increase in vagal activity, anti-pain, and anti-depression neurotransmitters (e.g., serotonin) as well as a decrease in stress hormones (Field 2012). On the one hand, acute exercise (i.e., cognitive examination shortly upon completing an exercise bout) is thought to enhance cognitive functioning by immediate neurochemical responses. For example, neurological effects of exercise as described by Lojovich (2010) and Knaepen et al. (2010) include increases in brain-derived neurotrophic factor, levels of synaptic proteins, glutamate receptors, and the availability of insulin-like growth factor, which altogether seem to contribute to cell proliferation and neural plasticity (Halperin et al. 2012). The cognitive enhancements are, furthermore, related to increased arousal and blood flow in the PFC (Lambourne and Tomporowski 2010). On the other hand, chronic exercise (i.e., cognitive examination after several weeks of regular physical exercise) could indirectly promote more permanently improved cognition and learning through morphological brain changes and improved cardio-respiratory functioning (Best 2010). This cognitive enhancement and improvements in the PFC are possibly cumulative and, therefore, lasting over time (Pesce 2009). Long-term cognitive benefits of regular cardio exercise are, furthermore, suggested to promote the accelerated cerebellar development in children (Bishop 2007) and the processes of healthier cerebrovascular aging, such as positive influences on blood flow, cell maintenance, and circulating catecholamines (Bolduc et al. 2013).

### Reviewing the effects of physical exercise in patients with ADHD

Seven prior literature reviews addressing the effects of physical exercise in children with ADHD have led to promising conclusions. Gapin et al. (2011) concluded in their review of six studies that physical exercise has both acute and chronic positive effects on behavioral and cognitive measures in children with ADHD. The authors suggested physical exercise as a potential supplement to medication. Archer and Kostrzewa (2012) reviewed three studies on this topic and reported a reduction of ADHD symptoms following moderate intensity cardio exercise, including impulse control, inattentiveness, stress, negative affect (e.g., depression), anxiety, and bad conduct. These reductions were related to an increased level of brain-derived neurotrophic factor, which is typically reduced in patients with ADHD. Two biopsychologically oriented reviews describing the relation between intense cardio exercise treatment and enhancement of cognitive and behavioral functioning of children with ADHD suggested positive influences on the structure, function, and growth of the brain as underlying mechanisms (Berwid and Halperin 2012; Halperin and Healey 2011). A review by Wigal and others (2013) on the effect of physical exercise on the physiology of childhood ADHD stressed that physical activity and stimulant medication both affect the dopaminergic and noradrenergic systems. One recently published review described ameliorated executive functions and social functioning and decreased levels of ADHD symptoms following the short-term aerobic exercise (Cerillo-Urbina et al. 2015). A final recently published review paper stated that especially mixed exercise programs appear beneficial for ADHD symptomatology and fine motor skills (Neudecker et al. 2015).

Despite the high informative value of the existing reviews, they present with several shortcomings. First, not all currently available studies on cognitive and behavioral gains of physical exercise in children with ADHD were considered so far as the field is rapidly expanding. Second, not all of the former reviews systematically categorized the specific types of exercise and outcome measures, preventing clear conclusions with regard to effectiveness and implementation (concerning for instance exercise type and frequency and expected advantages). Third, the value of the available reviews is limited, since information concerning the cognitive and behavioral outcome measures has not been considered sufficiently. Recently, the literature on this topic is growing, making frequent, critical and updated overviews necessary. The current literature review, therefore, aimed to provide a systematic, up-to-date overview on the effects of cardio and non-cardio exercise types on cognitive, behavioral/socio-emotional, and physical/(neuro)physiological outcome measures in children with ADHD, thereby also addressing the duration of these effects (acute or chronic). To better interpret and describe the existing literature, a quality screening of the included studies was performed. Furthermore, we described similar literature in adults with ADHD in an exploratory way, providing a more lifespan perspective on the effects of physical exercise in patients with ADHD.

## Methods

### Literature search

A literature search of scientific published literature in the databases of PubMed, PsycINFO and Web of Knowledge revealed 25 research articles published before April 2016 that described exercise in relation to cognition and behavior in children with ADHD and 4 articles related to this topic in adults with ADHD. The keywords “ADHD,” “children,” “adults,” “physical,” and “exercise” were combined with words related to physical exercise or outcome measures, such as “activity,” “sports,” “yoga,” “neuropsychology,” “cognition,” “executive,” “functioning,” and “treatment”. Only articles, including an ADHD group, describing some form of exercise and that were written in English, were included. In addition, relevant articles cited in the included papers were added. Through a systematic categorization of these articles, the outcomes were organized within a descriptive table (see Table 1). To provide additional grounds for comparison, effects sizes were calculated when possible (if not already presented in the original paper) by using Cohen’s *d* and its interpretation classification index (Cohen 1988), describing small effects (0.2 ≤ *d* < 0.5), medium effects (0.5 ≤ *d* < 0.8), and large effects (*d* ≥ 0.8). Effect sizes or ranges are shown in Table 1. However, there was unfortunately a profound variation in the characteristics of the study samples of the reviewed articles (e.g., medication use and control conditions), the type of functions assessed, the (un)availability of outcome statistics, the type of (neuropsychological) tests applied, and the comparisons performed (e.g., within or between subjects or mixed designs). We, therefore, deemed it unjustified to reliably compare effect sizes across studies.

**Table 1:** Sample characteristics and results of reviewed studies (*N* = 29)

Authors (year)	Group (*n* M:F)	Medication use in ADHD group (*n* dose; abstinence)	Age range (years)	Intervention type (duration)	Time of measurements relative to exercise	Outcomes (measures) [ES when reported]
Cardio exercise and acute effects in children						
Tantillo et al. (2002)	ADHD diagnosis (*DSM*-*III*; *n* = 18; 10M:8F) Healthy control group (*n* = 25; 11M:14F)	MPH (*n* = 18; dose 10–20 mg/2–4 times/day; no abstinence)	8–12	Exercise condition: treadmill exercise (personalized bouts; 5–25 min.) Control condition: rest (watching a video) Both groups underwent both conditions	Pre and directly post	*P/NP: Boys/girls ADHD vs. boys/girls control, post vs. pre:* Boys with ADHD: improved motor impersistence; increased spontaneous blink rate; decreased *Acoustic Startle Eye Blink Response (ASER)* latency. Girls with ADHD: decreased latency and increased amplitude of *ASER*. Unchanged: motor impersistence
Wigal et al. (2003)	ADHD diagnosis (Diagnostic interview schedule for children*; n* = 10; 10M:0F) Matched (gender and age) healthy control group (*n* = 8; 8M:0F)	Stimulant medication naïve	7–12	Cycle ergometer (varying intensity levels; 20–30 min.) No control condition	Pre and directly post	*P/NP: ADHD vs. control, post vs. pre:* No DA increase; lower lactate response; lower AC response; blunted increase of EPI and NE after physical exercise
Mahon et al. (2008)	ADHD diagnosis (parents reported previous diagnosis; *n* = 14; 14M:0F) No control group	MPH or amphetamine (*n* = 14; dose 12–42 mg/day; no abstinence)	9–12	Cycle ergometer (varying intensity levels; durations dependent on personal sub- maximal and peak effort) Within subjects: one exercise bout with and one bout without medication	During and directly post	*P/NP: MPH vs. No MPH during exercise:* (1) Sub-maximal exercise: higher heart rate. Similar: oxygen uptake, respiratory exchange ratio and perceived exertion; effects limited to cardiovascular functions. (2) Peak exercise: higher heart rate, oxygen uptake and work rate. Similar: oxygen uptake, respiratory exchange ratio and perceived exertion
Medina et al. (2010)	ADHD diagnosis (*DSM*-*IV; n* = 25; 25M:0F) MPH-users (*n* = 16) Non-MPH-users (*n* = 9)	MPH (*n* = 16; dose 5–30 mg/day; 48 h abstinence before exercise and tests)	7−15	Treadmill exercise (30 min.) Control condition: 1 min. stretching	Pre and directly post	*C: Post vs. pre:* Immediate improvements in speed and sustained attention and normalizations in impulsivity and vigilance (*Conners’ Continuous Performance Test; CPT*). Exercise effects comparable in medication users and non-users Unchanged: executive functions (*Digit Span, Coding B of WISC*-*R, Illinois Test of Psycholinguistic Abilities*—*Visual Sequential Memory*)
Chang et al. (2012)	ADHD diagnosis (*DSM*-*IV; n* = 40; 37M:3F), randomly assigned to Exercise group (*n* = 20; 19M:1F) Control group (*n* = 20; 18M:2F)	Stimulant medication (*n* = 20; information about type and dose not reported; no abstinence)	8–13	Exercise group: moderate intensity treadmill exercise (30 min.) Control group: watching running- related video (30 min.)	Pre and directly post	*C: Exercise vs. control, post vs. pre:* Improved inhibition in both groups (*Stroop Test; interference condition*) [ES: Cohen’s *d* = 0.28–1.26] Post-test performances of flexibility improved in exercise group but not in control group *(WCST; ‘non*- *perseverative errors’ and ‘categories completed’)* [ES: Cohen’s *d* = 0.34–0.74]
Pontifex et al. (2013)	ADHD diagnosis (suspected diagnosis, clinical status verified by a DSM-IV semi-structured interview; confirmed diagnosis verified by the ADHD Rating Scale IV; *n* = 20; 14M:6F) Matched (gender, age, pubertal status, SES) healthy control group (*n* = 20; 14M:6F)	Stimulant medication naïve	8–10	Exercise condition: treadmill exercise (20 min.) Control rest condition: seated reading Counter-balanced: both groups underwent both conditions	Directly post	*C: Exercise vs. control:* Improved in both groups: inhibitory control; allocation of attention resources; selective enhancement in stimulus classification; processing speed; reading comprehension; arithmetic. Unchanged: spelling (*Eriksen Flanker Test; Wide Range Achievement Test; neuroelectric assessment*) *P/NP: Exercise vs. control:* Improved in both groups: accurate response production and neurocognitive function (measured by ERPs)
Smith et al. (2013)*	At risk for ADHD (≥4 hyperactivity/impulsivity symptoms based on Disrupting Behavior Disorders Rating Scale*; n* = 14; 6M:8F) No control group	Stimulant medication naïve	5–9	Moderate-vigorous physical exercise such as moving around, hopping, running (30 min. daily; 8 weeks) No control condition	Pre and post (daily, weekly and after 9 weeks; assessment moment relative to physical exercise not reported)	*C: Post vs. pre:* Improvements: response inhibition and flexibility (*Shape School*-*set shifting*) Unchanged: planning (*Mazes subtest of the WPPSI*-*R*), spatial working memory (*Finger Windows*), verbal working memory (*Sentence Memory*), working memory and planning (*Numbers Reversed of the Woodcock*- *Johnson III Tests of Cognitive Abilities*) [ES: Cohen’s *d* = −0.10 to 0.43] Weekly measures: Both improved and unchanged levels of response inhibition (respectively *Red light/Green light* and *Simon Says*) [ES: Cohen’s *d* = −0.18 to 0.60] *B/SE: post vs. pre:* motor proficiency improved (*Bruin*-*Oseretsky Test of Motor Proficiency; BOT*-*2*); motor timing unchanged (*Motor Timing Task*) [ES: Cohen’s *d* = 0.96] Weekly measures: Behavior improvements based on teacher ratings (*Pittsburgh Modified CTRS*) [ES: Cohen’s *d* = 0.40–0.70] Daily measures: Behavior observations; improved ‘interrupting’ and ‘overall’ behavior; unchanged levels of ‘not speaking nicely’, ‘(un)intentional aggression’, ‘not following adult directions’ [ES: Cohen’s *d* = −0.18 to 0.78]
Hartanto et al. (2015)	ADHD diagnosis (clinical interview and Connor’s Total ADHD score >65; *n* = 26; 14M:12F) Healthy control group (*n* = 18; 5M:13F)	Stimulant medication (*n* = 15; information about type and dose not reported 24 h abstinence before exercise and tests)	10–17	Physical activity: intensity and frequency were measured in all participants by an actometer	Simultaneous assessment of activity and task performance on a trial-by-trial basis	*C: ADHD group vs. control group:* Between group: children with ADHD displayed better performance during intense physical activity on a cognitive control performance task *(Eriksen Flanker Paradigm)*, which was not found in the control group. *C: Within ADHD*-*group:* Higher intensity movements during correct trials and not during error trials; this difference did not exist in the control group A non-causal relation was described between cognitive control functioning and physical activity in children with ADHD specifically
Ziereis and Jansen 2015*	ADHD diagnosis (*ICD*-*10*; *n* = 39; 18M:5F) Randomly assigned to: exercise group 1 (*n* = 12; 9M:3F) Exercise group 2 (*n* = 11; 9M:2F) Wait-list control group (*n* = 16; males and females were mixed)	Stimulant medication naïve	7–12	Exercise group 1: Specific, moderate-vigorous physical exercises; focus on ball handling, manual dexterity and balance (60 min. weekly) Exercise group 2: Non-specific, moderate-vigorous physical exercises; no specific focus (60 min. weekly) Wait-list control group: no intervention *Time period: 12* *week intervention*	1-week pre, immediately following and 1-week post program	*C: Post vs. pre in both exercise groups:* Short-term effects (after first session): no support for improved executive functioning after any of the two types of physical exercise Long term positive effects (following 12 weeks of exercise): verbal working memory, digit span backward and letter-number-sequencing *(HAWIK*-*IV),* which were not found in the control group *P/NP: Post vs. pre in both exercise groups:* Long-term positive effects: motor performance and catching and aiming *(Movement*-*ABC)* Improvements were independent of exercise type and not seen in the control group
Cardio exercise and chronic effects in children						
McKune et al. (2003)	ADHD diagnosis (*DSM*-*III*-*R; n* = 19; 13M:6F), not randomly assigned to: Exercise group (*n* = 13; 10M:3F) Non-exercise control group (*n* = 6; 3M:3F)	MPH (*n* = 19; dose 10–30 mg/day, 1-2 times/day; no abstinence)	5–13	Various high-intensity exercise sessions (e.g., forms of running and jumping; 60 min.; 5 days a week) No control condition *Time period: 5* *weeks*	1-week pre, during and post (after 3 and after 5 weeks)	*B/SE: Post vs. pre:* Improved behavior; attention; emotional and motor skills, in both exercise and non-exercise groups; reportedly probably due to extra attention received (CPRS) Unchanged: task orientation and oppositional behavior
Gapin and Etnier (2010)	ADHD diagnosis (*diagnosed by medical professional; n* = 18; 18M:0F) No control group	Stimulant medication (*n* = 18; information about type and dose not reported; no abstinence)	9–12	Moderate to vigorous physical exercise (e.g., biking, skateboarding; 30-60 min. daily) No control condition. *Time period: 7* *days*	Pre and post (after 7 days)	*C: Post vs. pre:* Physical exercise was a significant predictor of planning performance *(ToL)* and a non- significant but trending predictor of other executive functions: inhibition, working memory, processing speed (*CPT*-*II; Digit Span WISC*-*III;* *Children’s Color Trial Test*)
Ahmed and Mohamed (2011)	ADHD diagnosis (*recruited from special needs school*; *n* = 84; 54M:30F), randomly and equally assigned to: Physical exercise group (*n* = 42; 27M:15F) Non-exercise control group *n* = 42; 27M:15F)	Information on medication intake not reported	11–16	Exercise group: aerobic, muscular and motor skills exercises (40 min. in first 4 weeks and 50 min. in last six weeks; 3 days a week) Control group: no physical exercise. Time period: 10 weeks Time period: 10 weeks	Pre and post	*B/SE: Post vs. pre:* Behavioral-cognitive and psychological functioning improved after physical exercise: three of five domains of a teacher report Behavior Rating Scale (based on *Connor’s Rating Scale*) improved significantly in the exercise group and not the control group: attention, motor skills and academic and classroom behavior Unchanged: task orientation, emotional behavior and oppositional behavior
Kang et al. (2011)	ADHD diagnosis (*diagnosis instrument not specified; n* = 28; 28M:0F), randomly assigned to Exercise group (*n* = 15; 15M:0F) Behavior education group (*n* = 13; 13M:0F) (*n* = 13; 13M:0F)	MPH (*n* = 28; dose 10–40 mg/day; no abstinence)	7–9	Exercise group: combination of running, goal- directed throwing, rope jumping and rest (90 min.; 2 days a week) Behavior education group: 12 education sessions on social behavior *Time period: 6* *weeks*	Baseline and post (after 6 weeks)	*C: Post vs. pre:* Improved in exercise and not in behavior education group: executive functions (e.g. flexibility); cognitive function and speed (*TMT*-*B; Digit Symbol of the Korean Educational Development Institute*-*WISC)* [ES: Cohen’s *d* = 0.18–0.49] *B/SE: Post vs. pre:* Improved in exercise and not in behavior education group: attention and social competencies (cooperativeness) Unchanged: hyperactivity; assertiveness; self-control (*ADHD Rating Scale; Social Skills Rating System*). [ES: Cohen’s *d* = −0.27 to 0.22]. Suggested mechanism: CA/DA/NE increases in the prefrontal cortex, nucleus accumbens and basal ganglia
Lufi and Parish-Plass (2011)	ADHD diagnosis (*DSM*-*IV*-*TR; n* = 15; 15M:0F) Other behavioral and emotional problems (*n* = 17; 17M:0F)	Stimulant medication naïve None of the participants used medication during the time of the study	8–13	Combined intervention of sports and behavioral techniques. The exercise intervention was comprised of vigorous physical exercise in individual sport such as relay races (30 min., weekly) and vigorous physical exercise in team sports such as soccer (40 min., weekly) No control condition *Time period: 1 academic year*	Pre, post (within 2 weeks following the intervention) and follow up (1 year after completion of the study)	*B/SE: Post vs. pre, acute post*-*test:* Following a sports and behavioral intervention, hyperactivity and behavior total scores as reported by participants and parents in both groups *(ASQ*-*P, YSR, CBCL*). Participants in both groups reported less anxiety, less somatic complaints, less internalizing behavior and less total problems. Parents of children in both groups reported less ADHD symptoms, aggression, anxiety, social problems, externalizing behavior and total problems. Unchanged: all other YSR and CBCL domains Sports + therapy = beneficial for both clinical groups, non-ADHD-specific. *B/SE: Post vs. pre, acute post*-*test:* Effects were maintained 1 year post-treatment. Especially anxiety consistently reduced from pretest, to posttest, to follow-up, reported by both children and parents
Verret et al. (2012)	ADHD diagnosis (*DSM*-*IV;* *n* = 21; 19M:2F) Physical exercise group (*n* = 10; 9M:1F; recruited from the same school) Control group (*n* = 11; 10M:1F; recruited from different areas)	Stimulant medication (*n* = 3 in physical exercise condition and *n* = 11 in control condition; information about type and dose not reported; no abstinence)	7–12	Exercise group: aerobic, muscular and motor skills exercises, cool down afterwards (45 min.; 3 days a week) Control group: no physical exercise. *Time period: 10* *weeks*	Pre and post (after 10 weeks)	*C: Exercise vs. control, post vs. pre:* Improved level of information processing, speed of visual search, auditory and sustained attention (*Test of Everyday Attention for Children*) *B/SE: Exercise vs. control, post vs. pre:* Parent- reported posttest improvements: total problems, social, thought and attention problems *(CBCL*; other scales unchanged). Teacher-reported posttest improvements: anxiety-depression; social problems (*CBCL;* other scales unchanged) *P/NP: Exercise vs. control, post vs. pre:* Better motor performance (increased locomotion and total motor skill scores)
Smith et al. (2013)*	At risk for ADHD (≥4 hyperactivity/impulsivity symptoms based on Disrupting Behavior Disorders Rating Scale; *n* = 14; 6M:8F) No control group	Stimulant medication naïve	5–9	Moderate-vigorous physical exercise such as moving around, hopping, running (30 min. daily; 8 weeks) No control condition	Pre and post (daily, weekly and after 9 weeks; assessment moment relative to physical exercise not reported)	*C: Post vs. pre:* Improvements: response inhibition and flexibility (*Shape School*-*set shifting*) Unchanged: planning (*Mazes subtest of the WPPSI*-*R*), spatial working memory (*Finger Windows*), verbal working memory (*Sentence Memory*), working memory and planning (Numbers Reversed of the Woodcock- Johnson III Tests of Cognitive Abilities) [ES: Cohen’s *d* = −0.10 to 0.43] Weekly measures: Both improved and unchanged levels of response inhibition (respectively *Red light/Green light* and *Simon Says*) [ES: Cohen’s *d* = −0.18 to 0.60] *B/SE: Post vs. pre:* Motor proficiency improved (*Bruin*-*Oseretsky Test of Motor Proficiency; BOT*-*2*); motor timing unchanged (*Motor Timing Task*) [ES: Cohen’s *d* = 0.96] Weekly measures: Behavior improvements based on teacher ratings (*Pittsburgh Modified CTRS*) [ES: Cohen’s *d* = 0.40–0.70] Daily measures: Behavior observations; improved ‘interrupting’ and ‘overall’ behavior; unchanged levels of ‘not speaking nicely’, ‘(un)intentional aggression’, ‘not following adult directions’ [ES: Cohen’s *d* = −0.18 to 0.78]
Chang et al. (2014)	ADHD diagnosis (*DSM*-*IV*-*TR; n* = 27; 23M :4F), non-random assignment to: Physical exercise group (*n* = 14; 10M:4F) Wait-list control group (*n* = 13; 13M:0F)	MPH (*n* = 7 in physical exercise condition and *n* = 6 in control condition; information about dose not reported; 24 h abstinence before tests)	5–10	Exercise group: moderate intensity water aerobic exercise and perceptual-motor water exercise and cool down afterwards (90 min.; 2 days a week) Wait-list control group Time period: 8 weeks	Pre and post (within 1 week of completing the study)	*C: Post vs. pre by group/condition:* Children with ADHD in the exercise group showed improved accuracy (Go/No-Go Task) with the no-go-stimulus (d = 0.9) as well as coordination of motor skills, whereas accuracy with the no-go-stimulus remained unchanged over time in the control group (*d* = −0.04). Physical exercise was therefore linked to increased levels of restraint/behavioral inhibition in children with ADHD Unchanged/reversed effect: go-stimuli scores were respectively longer and less accurate than in the control group, with no effect of time *P/NP: Post vs. pre:* The physical exercise group and not the control group achieved increased levels of hand-eye-coordination in throwing and bilateral hand-eye-coordination and dexterity (as measured by target throwing scores and bead moving scores of the *Basic Motor Ability Test*-*Revised*)
Choi et al. (2014)	ADHD diagnosis (*DSM*-*IV; n* = 30; 30M:0F), randomly assigned to: Physical exercise group (*n* = 17; 17M:0F) Education ADHD group (*n* = 13; 13M:0F) Age-matched healthy control group (*n* = 15; 15M:0F)	MPH (*n* = 35; dose 10–40 mg/day, 1 time/day; no abstinence as examining the relation of MPH with respectively physical exercise and behavioral education was one of the study aims	13–18	Exercise group: Aerobics exercises such as running and jumping rope (90 min.; several days a week) Education ADHD group: education sessions for behavior and control such as good behavior and bad behavior and interaction with family (50 min.; several days a week) Control group: no physical exercise Time period: 6 weeks	Pre and post (after 6 weeks of treatment)	*C: Post vs. pre:* Decreased scores on ADHD rating scale (*Dupaul ADHD rating scale, Korean version*) and decreased perseverative errors (*Wisconsin Card Sorting Test*) in the exercise group and not in the education group *P/NP: Sports ADHD group vs. education ADHD group:* Increased brain activity (fMRI) within the right frontal and temporal cortices during a Wisconsin Card Sorting Test stimulation Unchanged: activity within left parietal (trend), right middle temporal, right occipital, right parietal, right cerebellum and left middle temporal cortices The observed improvements in ADHD symptoms, perseverative errors and brain activity following physical exercise are proposed to be related to a higher MPH effectivity when medication is combined with sports
Pan et al. (2014)	ADHD diagnosis (DSM-IV; *n* = 24; 24M:0F), randomly assigned to: Training group (*n* = 12; 12M:0F) Non-training control group (*n* = 12; 12M:0F) Age and gender matched healthy control group (*n* = 24; 24M:0F)	Stimulant medication (*n* = 15; information about type and dose not reported; no abstinence)	7–14	Simulated developmental horse riding program in combination with moderate-vigorous fitness training (90 min., weekly) Control group: no physical exercise *Time period: 12* *weeks*	Pre and directly post	*P/NP: Post vs. pre by group comparison:* Both ADHD groups showed worse total motor proficiency scores than TD children; but the ADHD training group performed better than the ADHD non-training group at total motor proficiency, fine manual control, manual coordination, body coordination, strength and agility, manual dexterity and bilateral coordination (*Bruininks*-*Oseretsky Test of Motor Proficiency, second edition*) Non-significant: fine motor precision and integration, upper-limb coordination, balance, running speed and agility and strength *P/NP: Post vs. pre, within*-*group:* The ADHD training group showed improvements in all motor and fitness measures. The ADHD and TD non-training groups improved in some but not all motor skills or fitness measures In conclusion: children with ADHD thus exhibit low motor proficiency, cardiovascular fitness and flexibility at baseline, and these functions benefit from physical activity
Ziereis and Jansen (2015)*	ADHD diagnosis (*ICD*-*10*; *n* = 39; 18M:5F) Randomly assigned to: Exercise group 1 (*n* = 12; 9M:3F) Exercise group 2 (*n* = 11; 9M:2F) Wait-list control group (*n* = 16; males and females were mixed)	Stimulant medication naïve	7–12	Exercise group 1: Specific, moderate-vigorous physical exercises; focus on ball handling, manual dexterity and balance (60 min. weekly) Exercise group 2: Non-specific, moderate-vigorous physical exercises; no specific focus (60 min. weekly) Wait-list control group: no intervention Time period: 12 week intervention Time period: 12 weeks	1-week pre, immediately following and 1-week post program	*C: Post vs. pre in both exercise groups:* Short-term effects (after first session): no support for improved executive functioning after any of the two types of physical exercise Long term positive effects (following 12 weeks of exercise): verbal working memory, digit span backward and letter-number-sequencing *(HAWIK*-*IV),* which were not found in the control group. *P/NP: Post vs. pre in both exercise groups:* Long-term positive effects: motor performance and catching and aiming *(Movement*-*ABC)* Improvements were independent of exercise type and not seen in the control group
Janssen et al. (2016a)	ADHD diagnosis (*DSM*-*IV*-*TR;* *n* = 81), randomly assigned to: Physical exercise group (*n* = 27; 21M:6F) Neurofeedback group (*n* = 29; 21M:8F) Medication group (*n* = 25; 19M:6F)	Stimulant medication naïve (all patients had been stimulant medication free for at least 1 month before the beginning of the study) Medication group: MPH (*n* = 25; doses 5–15 mg/day)	7–13	Exercise group: Moderate -vigorous physical activity (not specified). The maximum heart rate (HRmax) was assessed. The aim was to first elevate the heart rate to 70-80 % of HRmax (5 × 2 min.) and then to 80-100 % of HRmax. (45 min. with 20 min. of effective training; 3 days a week) Neurofeedback group: Participants received theta/beta training with the aim of inhibiting theta (4-8 Hz) and strengthening beta (13-20 Hz) at Cz. (45 min. with 20 min. of effective training; 3 days a week) Medication group Time period: ~10 weeks	1 week pre and **~**1 week post	*P/NP: Group comparison, post vs. pre:* Children with ADHD (often showing increased slow wave activity –theta– and decreased fast wave activity –beta– in EEG studies which is linked to ADHD symptoms) displayed reduced theta power waves after neurofeedback and medication interventions but not after physical activity on an EEG-analysis. The medication and neurofeedback groups showed reduced theta power post-treatment during a resting condition. Solely the medication group displayed this reduction in an effortful task condition
Janssen et al. (2016b)	ADHD diagnosis (*DSM*-*IV*-*TR;* *n* = 81), randomly assigned to: Physical exercise group (*n* = 27; 21M:6F) Neurofeedback group (*n* = 29; 21M:8F) Medication group (*n* = 25; 19M:6F)	Stimulant medication naïve (all patients had been stimulant medication free for at least 1 month before the beginning of the study) Medication group: MPH (*n* = 25; doses 5–15 mg/day)	7–13	Exercise group: Moderate -vigorous physical activity (not specified). The maximum heart rate (HRmax) was assessed. The aim was to first elevate the heart rate to 70-80 % of HRmax (5 × 2 min.) and then to 80–100 % of HRmax. (45 min. with 20 min. of effective training; 3 days a week) Neurofeedback group: participants received theta/beta training with the aim of inhibiting theta (4–8 Hz) and strengthening beta (13–20 Hz) at Cz. (45 min. with 20 min. of effective training; 3 days a week) Medication group Time period: ~10 weeks	1 week pre and **~** 1 week post	*P/NP: Group comparison, post vs. pre:* The medication group displayed a specific post-intervention P3 amplitude increase, related to response inhibition. Across all groups, N2 amplitude increased from pre to post intervention. Thus, medication but not neurofeedback or physical exercise, was related to improved response inhibition
Non-cardio exercise and acute effects in children						
Hernandez-Reif et al. (2001)*	ADHD diagnosis (*DSM*-*IV; n* = 13; 11M:2F) No control group	Information on medication intake not reported	13–16	Physical exercise: Tai Chi, training of postures (30 min.; 2 days a week) for 5 weeks; then a non-exercise follow-up phase without Tai Chi (2 weeks) No control condition Time period: 5 weeks	Two weeks pre (baseline), directly post (after 5 weeks) and follow-up (after 7 weeks)	*B/SE: Post vs. pre:* Both acute (directly post) and chronic (2 weeks after intervention) effects were found; less anxiety, improved conduct, less daydreaming, less inappropriate emotions and less hyperactivity (*CTRS*) Unchanged: asocial behavior
Azrin et al. (2006)	ADHD diagnosis (*diagnosis instrument not described;* *n* = 1; M) No control group	Stimulant medication naïve	4	Gymnastic playground activities (i.e. sliding, climbing, swinging; 1 min.; recurring over 5 days) No control condition	Pre and directly post	*B/SE: Post vs. pre*: Improved observed attentive sitting in the classroom (observed by 2 school assistants) Physical exercise was however suggested to be a reinforcement. Substantial limitations were reported
Taylor and Kuo (2009)	ADHD diagnosis (*professionally diagnosed by a physician, psychologist or psychiatrist; n* = 17; 15M:2F) No control group	Information on medication intake not reported; children normally taking stimulant medication were asked to postpone intake to after the intervention	7–12	Walking, alternatingly in a park and 2 urban settings (respectively a downtown and a residential area; 20 min. per walk). Test administrators were blind to the walking condition No control condition	Directly post	*C: Walk in park vs. walk in urban* *settings:* Improved concentration (*Digit Span Backwards*); suggested greater influence of environment than physical exercise [ES park–urban settings: Cohen’s *d* = 0.52–0.77] Substantial limitations were reported
Non-cardio exercise and chronic effects in children						
Hernandez-Reif et al. (2001)*	ADHD diagnosis (*DSM*-*IV; n* = 13; 11M:2F) No control group	Information on medication intake not reported	13–16	Physical exercise: Tai Chi, training of postures (30 min.; 2 days a week) for 5 weeks; then a non-exercise follow-up phase without Tai Chi (2 weeks) No control condition Time period: 5 weeks	Two weeks pre (baseline), directly post (after 5 weeks) and follow-up (after 7 weeks)	*B/SE: Post vs. pre:* Both acute (directly post) and chronic (2 weeks after intervention) effects were found; less anxiety, improved conduct, less daydreaming, less inappropriate emotions and less hyperactivity (*CTRS*) Unchanged: asocial behavior
Maddigan et al. (2003)	ADHD diagnosis (*DSM*-*IV; n* = 10; gender not reported), randomly assigned to: Yoga/exercise group (*n* = 3) Massage group (*n* = 3) Control group (*n* = 4)	Stabilized on medication (information about type and dose not reported)	School-age (details not reported)	Exercise group: sessions of yoga exercise (6-20 min.; once a week for 6 weeks) Massage group: massages (6-20 min.; once a week for 6 weeks) Control group: no intervention *Time period: 12* *weeks*	Pre, during and post (after 0, 6 and a follow up at 12 weeks)	*C & B/SE: Post vs. pre in exercise group*: Improved ability to do homework; to cope in stressful situations; balancing; flexibility and concentration (observations and *CPRS*). Positive overall responses in both exercise and massage groups. Substantial limitations were reported
Jensen and Kenny (2004)	ADHD diagnosis (*DSM*-*IV and CPRS; n* = 19; 19M:0F) Yoga group (*n* = 11; 11M:0F) Control group (*n* = 8; 8M:0F) Randomly allocated to yoga group with the option to crossover after the first 20 sessions	MPH (*n* = 17; dose 15–40 mg/day; no abstinence)	8–13	Yoga group (60 min.; 20 sessions) Control group: cooperative games (social activities) Time period: 20 weeks	Pre and post (after 20 weeks)	*B/SE: Post vs. pre*: Improvements in both groups: perfectionism; DSM-IV hyperactive/impulsive; DSM-IV total. Yoga group improved scales (*CPRS*): oppositional; emotional liability; total; restless/impulsive; ADHD behavior Control group improved scales (*CPRS*): hyperactivity; anxious/shy; social problems. No significant differences according to teachers (*CTRS*) Substantial limitations were reported
Preliminary findings on the effects of physical exercise on cognitive and behavioral functioning in adults						
Fritz and O’Connor (2016) *Cardio exercise* *Acute effects*	ADHD screening diagnosis (*symptoms of* *adult ADHD* *assessed by the* *Adult ADHD Self*- *Report Scale;* *n* = 36; 36M:0F)	Stimulant medication naïve	18–33	Exercise condition: Cycling at 65 % VO2 peak (20 min.) Non-exercise control condition: Seated rest (20 min.) Within study design: all participants underwent both conditions	Baseline, Post 1, Post 2 (both immediately after the intervention)	*C/NP: Exercise condition vs. resting condition by time:* No significant improvements in sustained attention *(Continuous Performance Task and the Bakan Vigilance Task for sustained attention; Simple Reaction Time Task for psychomotor speed)* *B/SE: Exercise condition vs. resting condition by time:* Enhanced motivation to complete mental work in the exercise group over time (as assessed with a *Visual Analog Scale* with motivation indicators); significantly higher self-reported vigor, lower confusion scores, lower fatigue and lower depression scores in the exercise condition (*Profile of Mood States*-*Brief Form*) Unchanged: hyperactivity (*in the legs; Bakan test*), tension and anger Authors proposed that the increased vigor after physical exercise may be due to a similar working mechanism as described in stimulant medication, i.e. a DA-increase
Abramovitch et al. (2013) *Cardio exercise* *Chronic effects*	ADHD diagnosis (*DSM*-*IV; n* = 30;30M:0F) High physical activity group (*n* = 10; 10M:0F) Low physical activity group (*n* = 20; 20M:0F)	Stimulant medication naïve	Mean age = 27.29, SD = 5.87 (Infor-mation on age range not reported)	Physical exercise was assessed by a physical exercise subscale of a leisure time activity questionnaire, measuring exercises with a strong aerobic component (e.g., jogging; competitive sports; ≥ 30 min.) Based on the questionnaire, participants were divided into 2 groups: “high physical activity” and “low physical activity”	Correlational study, no intervention	*B/SE: High physical activity group vs. low physical activity group:* ADHD patients who frequently engage in physical exercise showed a healthier psychological well-being than ADHD patients who do not often exercise actively. They reported less meta-worrying, less problems with intrusive thoughts (i.e. difficulty removing them; thoughts of sadness and fear) and less behavioral impulsivity (as measured by the *Anxious Thoughts Inventory; Eysenk’s Impulsivity*-*Venturesomeness*-*Empathy Questionnaire; Distressive Thoughts Questionnaire*) Similar across groups: social and health worrying, venturesomeness, frequency of thoughts, thoughts of disapproval
Fuermaier et al. (2014a) *Non*-cardio exercise Acute effects	ADHD diagnosis (*clinical interview based on DSM*-*IV; n* = 17; 8M; 9F) Healthy adults (*n* = 83; 40M:43F)	MPH (*n* = 4; dose 10–72 mg/day, 1 time/day; no abstinence)	18–31	Passive exercise group: Passive Whole Body Vibration (WBV) was implemented, participants were sitting on a chair that was ascended on a vibrating platform (*Vibe 300*, Tonic Vibes, Nantes, France) Control condition: Resting period without vibration Procedure: The intervention consisted of four 2-min treatments of vibration (vibration condition) and four 2-min trials of resting (control condition) for each participant	Directly post	*C* *: Vibration vs. resting:* WBV significantly improved attention performance (*Stroop Color Word Interference Test*) in both groups, with a small effect size in the healthy control group (*d* = 0.44) and a medium effect size in the ADHD group (*d* = 0.68). Passive physical exercise was thus related to improved cognitive functioning in healthy adults and adults with ADHD
Fuermaier et al. (2014b) Non-cardio exercise Acute and chronic effects	ADHD diagnosis (*DSM*-*IV; n* = 1; M) Control group (*n* = 6; 3M:3F)	MPH (dose 10 mg, 4 times/day; no abstinence)	20–25	Passive exercise—ADHD patient: Passive WBV was applied (15 min.; 3 times a day on 10 consecutive days), the participant was sitting on a chair that was mounted on a vibrating platform (*Vibe 300*, Tonic Vibes, Nantes, France) Control group: assessed with the same test battery to control for practice effects; no WBV Time period: 10 days	1 day pre, 16 h post and follow-up (after 12 days and after 25 days-treatment started on day 2)	*C: Post vs. pre:* Descriptive performance data showed 1) in healthy participants: improved vigilance, flexibility, working memory and inhibition after repeated assessments; 2) in the ADHD patient: improved alertness, divided attention, vigilance, flexibility, inhibition, divergent thinking, verbal fluency and self-reported impairments of attention (especially from first to second assessment). WBV-treatment was therefore related to acute cognitive gains Unchanged: alertness reaction times, distractibility and working memory Follow-up: most of the assessed cognitive functions returned to their pre-experiment-level (e.g. flexibility, inhibition, vigilance and self-reported impairments of attention)

AC, acetylcholine; ADHD, attention–deficit/hyperactivity disorder; AVL, ADHD *Vragenlijst;* ASRS, Adult ADHD Self-Report Scale; B/SE, behavioral/socio-emotional; CA, catecholamines; C, cognitive; CBCL, Child Behavior Checklist; CPRS, Connor’s Parents Rating Scale; CPT-II, Connor’s Continuous Performance Test; CTRS, Connor’s Teacher Rating Scale; DA, dopamine; EPI, epinephrine; ES, effect size(s); F, female; HRmax, maximum heart rate; ICD-10, International Statistical Classification of Diseases and Related Health Problems; M, male; MPH, methylphenidate; NE, norepinephrine; P/NP, physical/(neuro)physiological; post, measurement after exercise; pre, measurement prior to exercise treatment; SES, social economic status; TMT, Trail Making Test; ToL, Tower of London; WBV, whole body vibration; WCST, Wisconsin Card Sorting Test; WISC-R, WISC-III, Wechsler Intelligence Scale for Children; WPPSI-R, Wechsler Preschool and Preliminary Scale of Intelligence–Revised; *, study also mentioned in another section

### Analysis

To provide a systematic overview of the existing literature, categorizations were made for three variables; exercise type (cardio versus non-cardio), duration of effects (acute versus chronic), and outcome measures (cognitive, behavioral/socio-emotional, and physical/(neuro) physiological).

Acute effects of exercise were defined as the effects of physical exercise immediately after the workout, with a maximum of 24 h; hence, the outcome measures stemmed from the same full day as the exercise intervention. Chronic effects of exercise were defined as outcomes lasting longer than 24 h after the exercise intervention, with assessments after one to 10 weeks, depending on the follow-up period of included studies. This classification into acute and chronic effects was made, because physical aftereffects of exercise were thought to last for the first full day but to diminish after a resting period during the night. Persisting effects after nocturnal rest and recovery are considered to be long-lasting.

Cardio exercise included all types of workout that lead to an increased heart rate and oxygen use and that are performed for a relatively long duration, such as (treadmill) running, (ergo meter) cycling, swimming, and jumping. Any exercise type that is performed at a lower energy level and does not intensely increase the heart rate was classified as non-cardio exercise, including yoga, walking, and playground activity.

Outcome measures of the reviewed papers were classified into one of three categories, namely, “cognitive outcome measures” [including intelligence scores and (neuropsychological) tests for attention, planning, inhibition and memory], “behavioral and socio-emotional outcomes” (comprising parent and/or teacher questionnaires on the behavioral functioning of children, e.g., ADHD symptoms), and “physical and (neuro)physiological outcomes” (e.g., sheer physical/physiological effects).

Finally, we screened the included papers for their methodological quality to weigh the descriptions of the studies and the conclusions of this review. Two independent raters classified the following four important quality determinants of treatment studies as adequate (A), inadequate (IA), not applicable (NA) or not reported (NR). First, ADHD-diagnosis was assessed by standardized measures (e.g., DSM, ICD, ARS, Connor’s rating scale) to diagnose or operationally define behaviors and symptoms of participants. Second, sample size: for detecting a medium effect size (*f* = 0.25) in the most commonly employed design of the studies included in this review (a within-between group interaction in a repeated measures ANOVA with two groups (e.g., ADHD versus control) and one within-subjects variable (e.g., pre-measurement versus post-measurement), 17 participants are needed per group (with a power of 0.80 and an alpha of 0.05). When more within-subjects variables are added, a fewer participants are necessary (e.g., adding a low versus high intensity exercise condition reduces the needed participants to 12 per group) but when a control group is omitted, more participants are necessary to demonstrate a pre–post effect (34 participants are needed). Third, control condition/group: either a between group or a within subject comparison was made comparing exercise to some other condition without exercise. Last, control for medication use: either all participants were on medication, naïve for medication or off medication during the treatment/control condition, or it was checked whether medication influenced the results (e.g., by comparing subgroups).

## Results

### Cardio exercise: acute effects

After cardio exercise (e.g., treadmill running, cycling), several positive effects on (higher) cognitive functioning of children with ADHD were found. Response inhibition, cognitive control, attention allocation (based on event-related brain potentials; Pontifex et al. 2013), cognitive flexibility, processing speed, and vigilance were found to be improved in children with ADHD when assessed immediately after physical exercise (Chang et al. 2012; Hartanto et al. 2015; Medina et al. 2010; Pontifex et al. 2013; Smith et al. 2013). Academic performance was also found to be improved in both children with ADHD and healthy children following physical exercise, including enhanced levels of reading comprehension and arithmetics (Pontifex et al. 2013). However, not all assessed aspects of academic and cognitive functioning were sensitive to the effects of physical exercise, with unchanged performance levels of verbal short-term and working memory, sub-indexes of executive function performances (e.g., perseverative responses), spelling, reading, and color naming (Chang et al. 2012; Medina et al. 2010; Pontifex et al. 2013; Smith et al. 2013; Ziereis and Jansen 2015).

With respect to behavioral and school-day functioning, parents and teachers reported children with ADHD to display overall improvements after physical activity (e.g., diminished interruption and unintentional aggression). No effects, however, were found concerning intentional aggression, language use and following directions (Smith et al. 2013). The authors did not report whether the raters were blind to the treatment of the child.

Finally, regarding physical/(neuro)physiological acute effects of cardio exercise, improved motor impersistence (defined as the inability to sustain motor acts, such as keeping the mouth open, protruding the eyes, centrally fixing the eyes or shutting the eyelids) was reported in boys with ADHD. This effect was, however, not found in girls, lacking a clear explanation (Tantillo et al. 2002). Pontifex et al. (2013) described physical exercise-induced improvements in neurocognitive function and response production as measured by event-related potentials (ERPs), cautiously linking this to behavioral and academic gains. Wigal et al. (2013) demonstrated abnormal physiological after effects of physical exercise in children with ADHD (including more blunted (nor)epinephrine responses and no dopamine increase) which are in contrast to the effects found in healthy controls. Another study stressed that children with ADHD off MPH showed different, attenuated physiological responses to exercise compared to children with ADHD on MPH, with the latter having a higher sub-maximal heart rate during exercise (Mahon et al. 2008). However, other cardiorespiratory measures and perceived exertion were found to be unaffected by the use of MPH (presumably due to the small sample size (*n* = 14); Mahon et al. 2008).

### Cardio exercise: chronic effects

Cardio exercise (e.g., running and jumping) has also been linked to longer lasting effects on cognition in children with ADHD, resulting in improved attention (including auditory sustained attention and selective attention/information processing), executive functioning (including set shifting, (accuracy of) response inhibition and planning), verbal working memory, and cognitive speed (Chang et al. 2014; Choi et al. 2014; Gapin and Etnier 2010; Kang et al. 2011; Smith et al. 2013; Verret et al. 2012; Ziereis and Jansen 2015). However, a lack of robustness of chronic effects on cognition after cardio exercise is shown by some studies not reporting affected functions [e.g., (working) memory], and by studies not confirming significant beneficial long-term effects in the areas of inhibition, processing speed, planning, memory span, and continuous motor timing (Gapin and Etnier 2010; Smith et al. 2013). The lack of agreement in findings might partly be explained by the small sample sizes examined in the latter two studies (*n* = 14 and *n* = 18, respectively, which is less than the requested *n* = 34 for a one-factorial within subject design) and their consequential low statistical power.

A number of studies provided ample evidence for chronic effects of cardio exercise on parent or teacher ratings and observations of a broad range of behavioral and socio-emotional outcomes. Overall functioning, general ADHD symptomatology, attention, the child’s self-esteem, anxiety, depression, somatic complaints, academic and classroom behavior, and social behavior were reported to be improved after cardio exercise, extending beyond the day on which the physical exercise took place (Ahmed and Mohamed 2011; Choi et al. 2014; Kang et al. 2011; Lufi and Parish-Plass 2011; Smith et al. 2013; Verret et al. 2012). Social behavior in this regard refers to general social skills, social competency, cooperativeness, perceived social, thought and attention problems, interrupting behavior and (unintentional) aggression. No consistent evidence was found in these studies, however, for long-term improvements of hyperactivity, assertiveness, task-orientation, self-control, emotional behavior, and oppositional/defiant social behavior (e.g., language use, intentional aggression, not following adult directions, and teasing). The fact that effects were not significant is presumably (partly) explained by design limitations (e.g., lack of a control group or small sample size; Kang et al. 2011; Smith et al. 2013). In line with this lack of robustness, McKune et al. (2003) presented comparable improvements in both the physical exercise and the control group with regard to attention, behavior, and emotional skills. These effects were thought to be (partly) explained by the extra attention children received in both conditions.

Last, motor performance scores, including locomotion, hand–eye coordination, and general motor skills (e.g., fine motor skills, strength, and agility) were found to benefit from physical exercise (Chang et al. 2014; McKune et al. 2003; Pan et al. 2014; Verret et al. 2012; Ziereis and Jansen 2015). Although Pan and colleagues (2014) described overall improvement in motor skills and coordination, performance of some motor skills (e.g., balance and running speed) improved comparably in children with ADHD who performed physical exercise and a control group without exercise, which points to test–retest effects (Pan et al. 2014). Regarding neurophysiological improvements, Choi et al. (2014) found in a functional magnetic resonance imaging study that brain activity enhanced within the right frontal and temporal cortices during a set-shifting task, but that brain activity within several other cortices remained unchanged. An EEG/ERP study showed inconsistent findings, with an increased No-Go N2 amplitude but no increased No-Go P3 amplitude (which are linked to improved executive function) in a physical activity group after 10 weeks of exercise, and no reduction in EEG theta power (which is often increased in children in ADHD, and are associated with more ADHD symptoms; Janssen et al. 2016a, b).

### Non-cardio exercise: acute effects

The number of studies on acute effects of non-cardio exercise is scarce, and their conclusions are all limited by inadequate study designs that were justly addressed by the authors of these studies (e.g., small sample sizes, absence of a control group/condition). One study reported improved anxiety and conduct as well as less hyperactivity, inappropriate emotions and daydreaming in children with ADHD following tai chi sessions, but asocial behavior remained unchanged (Hernandez-Reif et al. 2001). Another study showed that although the attention of children with ADHD significantly improved after a walk in the park, it remained unclear whether this effect could be attributed to the physical activity or the natural environment (Taylor and Kuo 2009). A case study stated that playground activity appeared to diminish hyperactive behavior, but this improvement might (partly) be due to the reinforcing and appraising character of the situation rather than the physical activity itself (Azrin et al. 2006).

### Non-cardio exercise: chronic effects

Similar to the acute effects of non-cardio exercise, information about the chronic effects of non-cardio exercise is scarce. The enhanced behavioral/socio-emotional measures as described by Hernandez-Reif et al. (2001) remained on a long-term basis as well. Maddigan et al. (2003) described positive effects of yoga on several general cognitive and neuropsychological functions (i.e., the ability to do homework and to cope with stressful situations, balancing, flexibility, and attention). This study, however, claimed that solid conclusions cannot be drawn from the results, because of the small sample size included (*N* = 10) as well as methodological limitations related to the study execution (e.g., unstable attendance to experimental sessions). Another study also reported improvements of behavior and emotions following yoga (e.g., in terms of ADHD symptomatology and social behavior; Jensen and Kenny 2004). The underlying behavior observation ratings were, however, ambiguous, with more positive ratings of teachers than parents, possibly due to the fact that teachers were observing the children, while they were under the influence of stimulant medication. Furthermore, as shown by behavioral improvements in the social intervention control group, positive effects were not limited to the yoga group. Finally, the sample size included in this study was too small for sufficient power (*N* = 19, Jensen and Kenny 2004).

### Preliminary findings on the effect of physical exercise on adults with ADHD

Although the literature on the effect of physical activity on (cognitive) functioning in adults remains particularly scarce to date, a number of studies presented promising results. Two studies described a positive relation between engaging in physical exercise and behavioral/socio-emotional outcomes, including enhanced levels of self-reported motivation and vigor, and lower levels of impulsivity, worrying, intrusive and worrisome thoughts, confusion, fatigue and depression (Abramovitch et al. 2013; Fritz and O’Connor 2016). The findings were not robust for all measures, as Fritz and O’Connor (2016) failed to find improvements of hyperactivity or cognition despite examining a sample of adequate size (*N* = 36).

With regard to non-cardio physical activity, Fuermaier et al. (2014a, b) reported promising effects of Whole Body Vibration (WBV) on cognitive functioning in adults with ADHD. WBV is a passive exercise method in which people are physically activated by exposure to environmental vibration by sitting or standing on a vibrating plate. In a group study, acute effects of WBV treatment on attention performance were found, with larger beneficial effects in adults with ADHD (medium effect size) than healthy adults (small effect size; Fuermaier et al. 2014a). It seems noteworthy to point out that these effects could be observed after a WBV treatment of only a few minutes. A case study about a young male adult with ADHD who underwent WBV treatment during 2 weeks, described long-lasting acute effects (assessed after nocturnal rest, within 24 h following the two-week intervention) on cognition (e.g., inhibition, flexibility, self-reported levels of attention). However, since most of the cognitive measures returned to their baselines levels after 2 weeks of no WBV, chronic effects were not found. The acute effect of WBV was substantially larger than the test–retest effect in a healthy control group receiving no treatment (*N* = 6). Reaction times, distractibility, and working memory were unaffected by WBV treatment.

### Methodological quality screening of the included studies

Table 2 displays the classification of four important methodological quality determinants of the reviewed studies. As shown in Tables 1 and 2, most studies adequately described the diagnosis of the included patients with ADHD (23 out of 29 studies). Almost half of the studies were, however, underpowered because of too small sample sizes (13 out of 27 studies; 2 studies were case reports). Although the majority of the studies adequately included and described a control condition/group (18 out of 29 studies), a substantial share of the papers did not include an adequate control condition/group without exercise. Most of the papers reported stimulant medication use of the included patients, and sufficiently described whether patients took their regular medication, were medication naïve, whether medication use was abstained during the experiment, or whether medication influences were controlled for in the analysis (22 out of 27 studies). Overall, it can be concluded that especially small sample sizes and the absence of adequate control conditions/groups were often limiting the reviewed studies.

**Table 2:** Number of studies (k) screened for adequate and inadequate quality of four relevant methodological quality indicators

Quality indicators	Adequate	Inadequate	Not reported	Not applicable
1. Diagnosis assessed by standardized measures	23	1	5	0
2. Sample size	14	13	0	2
3. Control condition/group	18	11	0	0
4. Control for medication use	22	5	2	0

## Discussion

In this paper, the literature on the potential acute as well as chronic effects of both cardio and non-cardio physical exercise on cognitive, behavioral/socio-emotional, and physical/(neuro)physiological outcome measures in patients with ADHD was reviewed.

First, cardio exercise in children with ADHD appeared to be related to acute positive changes in a wide variety of these outcome measures with effect sizes (when reported) up to 1.26 (Cohen’s d). However, findings were selective, with some outcome measures showing an improvement following exercise and with other measures showing no improvement. Second, studies on chronic effects following cardio exercise implied that children with ADHD also benefit on a longer term (i.e., after a substantial period of rest) from cardio physical activity as indicated by significant improvements with effect sizes (when reported) up to 0.96 (Cohen’s d). Selective effects were observed here as well by some measures not showing improvements. It should be considered that some null findings may have been caused by inadequate research designs (see Results section). Several reported differences that did not reach significance were in the expected direction. Although most studies on cardio exercise generally found improvements on cognitive functions that are often impaired in ADHD, such as speed of information processing, attention, inhibition, and flexibility (Ahmed and Mohamed 2011; Chang et al. 2012; Chang et al. 2014; Choi et al. 2014; Gapin and Etnier 2010; Hartanto et al. 2015; Kang et al. 2011; McKune et al. 2003; Medina et al. 2010; Pontifex et al. 2013; Smith et al. 2013; Verret et al. 2012; Ziereis and Jansen 2015), some studies pointed to a lack of robustness by not (consistently) revealing comparable improvements across studies (Chang et al. 2014; Gapin and Etnier 2010; Medina et al. 2010; Pontifex et al. 2014; Smith et al. 2013; Ziereis and Jansen 2015). For example, working memory and memory functions were found to be improved on the long term by exercise in one study (Ziereis and Jansen 2015) but not in others (Gapin and Etnier 2010; Medina et al. 2010; Smith et al. 2013). This inconsistency in findings may be related to differing methodological study qualities, which will be further discussed under ‘limitations.’ Overall, it can be stated that the reviewed studies describe acute as well as chronic beneficial effects of cardio exercise on a wide variety of cognitive and behavioral functions in children with ADHD, but that cardio exercise does not necessarily result in functional improvement in all areas.

With regard to non-cardio exercise and acute effects, studies suggested cognitive, behavioral and motor improvements in children with ADHD, with effect sizes (when reported) up to 0.77. Drawing firm conclusions seems, however, premature, because of the third variables potentially explaining or moderating these effects (e.g., environmental factors, reinforcement and motivation) and because of the limited number of studies performed. Moreover, most studies were characterized by methodological weaknesses (e.g., small and heterogeneous groups, lacking control group/condition and logistical problems with study execution in home settings). Although some chronic beneficial effects were demonstrated for non-cardio exercise on general cognitive and behavioral functions, valid conclusions cannot be drawn either because of weak study designs or the absence of theoretical support provided for the findings. In the light of the limitations discussed, clear conclusions of the acute and chronic effects of non-cardio exercise on children with ADHD remain elusive to date. Based on our current knowledge, cardio exercise appears to be a more promising treatment method for children with ADHD than non-cardio exercise with regard to both acute and chronic cognitive and behavioral effects, but more well-designed studies are needed.

The literature on the effects of physical exercise in adults with ADHD also remains relatively scarce. In the available studies, beneficial effects (of medium size) of both active (leisure time sports activities with a strong aerobic component) and passive physical activity (WBV) were described to be related to improved cognitive and behavioral functions including attention, inhibition, motivation and impulsivity (Abramovitch et al. 2013; Fritz and O’Connor 2016; Fuermaier et al. 2014a, b). Not all assessed functions improved and the duration of the effects of passive physical remains to be elucidated, but the fact that positive findings were shown in these studies is promising when considering physical exercise interventions for adults with ADHD.

In view of the findings of this literature review, the question arises as to *why* physical exercise could improve cognitive and/or behavioral functioning in ADHD. As mentioned in the introduction, the underlying working mechanism of chronic exercise likely entails enhanced neural growth and development which may have long-term implications (Best 2010; Bishop 2007; Bolduc et al. 2013; Halperin et al. 2012; Pesce 2009). Especially when physical exercise is performed at young age, long-term positive responses of cell-proliferation might take place (as demonstrated in animal research; Halperin et al. 2012; Kim et al. 2004). Regular physical exercise may, therefore, be especially beneficial for children with ADHD, because cardio exercise increases specific catecholamines and proteins/enzymes that are typically reduced in ADHD (e.g., dopamine, tyrosine hydroxylase and brain-derived neurotrophic factor; Chang et al. 2012; Hattori et al. 1994; Kim et al. 2011). In addition, cognitive functions that have often been found impaired in patients with ADHD, such as executive functions, improved after acute exercise in the majority of the reviewed studies. According to Chang and colleagues (2012), this can be explained by an exercise-induced improved allocation of attention resources and by positive influences on the dorsolateral PFC. Interestingly, memory improvements after acute cardio exercise in healthy adults appear to be related to better-facilitated molecular mechanisms of memory encoding and consolidation (Roig et al. 2013).

It is assumed that physical exercise entails similar neurobiological effects as stimulants (e.g., increased availability of monoaminergic catecholamines in the brain, Fritz and O’Connor 2016; Wigal et al. 2013) and that these effects result in improved functioning in overlapping areas of cognition. A number of studies even showed (cognitive) gains of physical exercise on top of medication treatment (i.e., when children were on stimulant medication during the exercise bouts and tests; Choi et al. 2014; Gapin and Etnier 2010; Jensen and Kenny 2004; Kang et al. 2011; Maddigan et al. 2003; Mahon et al. 2008; McKune et al. 2003; Tantillo et al. 2002; Verret et al. 2012). Regular physical exercise could, therefore, be an effective (additional) treatment option for children with ADHD (in this review, exercise bouts of 30 min were most common with a range of 1–90 min). This would especially be beneficial in the following cases: (1) when deficits are not normalized by pharmacological treatment (e.g., see Tucha et al. 2006), (2) when pharmacological treatment is not the first choice treatment, (3) when effects of pharmacological treatment on a child’s problem behaviors are inconsistent (Swanson et al. 2011) or (4) when children suffer from milder disturbances which might benefit from physical exercise, thus possibly eliminating the need for pharmacological treatment. The advantages of physical exercise are broad: exercise can be combined with all treatment approaches currently applied in ADHD (including pharmacological treatment), it is cheap, non-invasive, and easy to implement, it has additional health benefits (e.g., potential prevention of chronic diseases and obesity), and can improve psychological well-being (e.g., Warburton et al. 2006). However, exercise programs should at all times be individually adapted and potential risks as well as physical and health issues have to be taken into consideration. Based on the demonstrated effectiveness and the practical implementation in everyday life, daily exercise bouts of 30 min appear reasonable.

### Limitations

Despite the existing evidence for effectiveness, the present findings ought to be interpreted with caution as the majority of described studies suffered from one or several methodological shortcomings. The methodological quality of the included papers was screened by classifying four important quality determinants for each paper (see Table 2). Although the majority of the studies adequately assessed the ADHD symptoms by means of standardized measures and controlled for the use of medication, almost half of the studies were underpowered, and one-third of the studies did not include an adequate control group/condition without exercise. We weighed these limitations in this review, and are therefore reluctant to draw conclusions about the beneficial effects of non-cardio exercise. However, a substantial amount of studies on the effects of aerobic exercise that did adopt rigorous methodological designs point to beneficial short- and long-term effects for patients with ADHD. This finding stresses the importance of developing exercise programs for children and adults with ADHD and of rigorously evaluating their effects by means of randomized controlled trials. By including adequate control groups/conditions, potential confounding variables can be ruled out, such as received attention, environmental effects as well as possible testing/learning effects of repeated administration of the same tests. Furthermore, none of the studies examining behavioral/socio-emotional outcomes reported whether raters were blind to the treatment condition of the participants. To overcome bias or a placebo effect, it is important for future studies that either raters are blind to the treatment conditions or that more objective assessments are used. Another factor influencing the results of the studies may be an unequal gender distribution in favor of boys. This, however, is not necessarily a weakness, since ADHD prevalence rates are higher in boys relative to girls (Gapin and Etnier 2010), but articles reporting gender differences in physical exercise effectiveness point to the need of equal numbers of boys and girls with ADHD in studies (e.g., Tantillo et al. 2002). Last, exercise bouts were generally not standardized but rather varying with regard to type, intensity, and duration. Although this is on the one hand a strength, since it shows effectiveness at varying and personalized intensities, it is on the other hand a weakness as it is not well controlled, making clear conclusions difficult.

In addition to these study-specific problems, there were some limitations on the review level as well. Reliably comparing studies and drawing general conclusions was complex, since studies differed considerably in many aspects (i.e., study designs, type of cognitive tests used, measurement outcomes, exercise types and duration and sample sizes), making direct comparison difficult. Furthermore, studies examined samples of children and adolescents with an age ranging from 5 to 18 years. Since symptomatology of ADHD and cognitive functions change with age and individual development (e.g., Schmidt and Petermann 2009), it would have been desirable to analyze whether specific age groups benefit more from certain forms of exercise (cardio versus non-cardio). However, this was not possible because of the small number of studies. This difficulty is even complicated by the fact that children of different age groups were pooled in the same samples (e.g., Medina et al. 2010: age range 7–15 years) which might even have reduced the impact of exercise on functioning. A final limitation on the review level is that, despite the growing research focus on alternative ADHD treatments, only a small number of studies are available.

### Future perspectives

Given the promising current body of evidence regarding the cognitive, behavioral/socio-emotional, and physical/(neuro)physiological effects of physical exercise in the treatment of ADHD symptoms, further research is necessary to substantialize these findings. It is of importance to clearly distinguish between, on the one hand, the exercise type (cardio versus non-cardio) and, on the other hand, the duration of the effects (acute versus chronic). This distinction could help establish the type of exercise that is most effective for treating ADHD symptoms in clinical practice. Even though especially cardio exercise appears promising, both cardio and non-cardio exercise should be further examined in well-controlled studies to allow more definite conclusions, e.g., by means of randomized-controlled trials. Future studies should further explore the potential treatment effects and implementation possibilities of physical exercise in patients with ADHD and would be advised to take the following issues into account. First, studies should consider multiple assessment moments by applying designs which allow the measurement of both acute and longitudinal effects. Second, studies should include adequate control groups/conditions without exercise to capture the true effect of exercise, and to control for confounding variables, such as the attention received or test–retest effects. In addition, more general aspects with regard to the methodological quality should be considered, including larger sample sizes, the use of a wide variety of cognitive, behavioral/socio-emotional and physical/(neuro)physiological outcome measures, the use of raters who are blind for the treatment condition, and controlled intensity and duration of the bouts of exercise. Control groups of healthy children or adults should be included to assess whether exercise induced improvements are (1) normalizing functioning of patients with ADHD and (2) whether improvements are comparable to the improvements described in healthy people. Finally, future studies should address the interaction of physical exercise and stimulant medication in a controlled design, examining possible complementary and/or differing effects.

Due to the promising findings and other advantages of physical exercise implementation (e.g., inexpensive, easy to apply, additional health benefits), future studies should focus on questions like “Are there responders and non-responders?,” “Do certain dysfunctions improve more by exercise than others?,” “What physical activity (e.g., running, yoga, etc.) has the best outcome for certain behaviors (e.g., cognition, social behavior)?,” “What is a realistic exercise plan (e.g., with regard to intensity, frequency, duration, etc.)?,” and “Does regular physical exercise have an additional effect to other treatments (e.g., stimulant drug treatment) on symptoms of ADHD?.” A reasonable method for daily implementation should be addressed by considering potential practical difficulties of physical exercise performance on, for instance, school and work days. In this light, passive exercise (e.g., whole body vibration) could also be considered as a practical and well-implementable form of exercise. Future studies should thus further explore both active and passive forms of exercise and their possibilities for implementation in the treatment of children and adults with ADHD.
